# Optic Flow: Perceiving and Acting in a 3-D World

**DOI:** 10.1177/2041669520987257

**Published:** 2021-02-03

**Authors:** Brian Rogers

**Affiliations:** University of Oxford, Oxford, United Kingdom

**Keywords:** 3-D perception, cue combination, depth, experience/learning/expertise, memory, motion, navigation/wayfinding, optic flow, perception, perception/action

## Abstract

In 1979, James Gibson completed his third and final book “The Ecological Approach to Visual Perception”. That book can be seen as the synthesis of the many radical ideas he proposed over the previous 30 years – the concept of information and its sufficiency, the necessary link between perception and action, the need to see perception in relation to an animal's particular ecological niche and the meanings (affordances) offered by the visual world. One of the fundamental concepts that lies beyond all of Gibson's thinking is that of optic flow: the constantly changing patterns of light that reach our eyes and the information it provides. My purpose in writing this paper has been to evaluate the legacy of Gibson's conceptual ideas and to consider how his ideas have influenced and changed the way we study perception.

## Background

It is over 40 years since James Gibson published his final book: “The Ecological Approach to Visual Perception" and therefore an appropriate time to evaluate the significance of his approach and ideas. To set his achievements in context, it is important to point out that there have been few individuals in the field of perception whose ideas have provoked as much controversy as those of J. J. Gibson. In 1991, Stuart Sutherland wrote:

“*Gibson did for perception what Skinner did for animal learning: he handicapped a generation of workers by his blinkered and oversimplified approach*”.

In contrast, Edward Reed (1998) wrote that:

“*whatever the final status of Gibson's theories, his work has significantly changed the scientific study of human awareness and has deep implications for anyone interested in human behaviour and knowledge*.” (p. 2)

One of the most significant differences between traditional theories of perception and Gibson’s ideas concerns the availability of information. Ignoring for the moment the issue of how we should understand the word “information,” it has often been assumed that the information reaching our senses is insufficient to explain the richness of our perceptions. Take, for example, what Richard Gregory wrote on the first page of his 1966 book “Eye and Brain”:

We are given tiny distorted upside-down images in the eyes, and we see separate solid objects in surrounding space. From the patterns of stimulation on the retinas we perceive the world of objects, and this is nothing short of a miracle.

**Figure 1.**
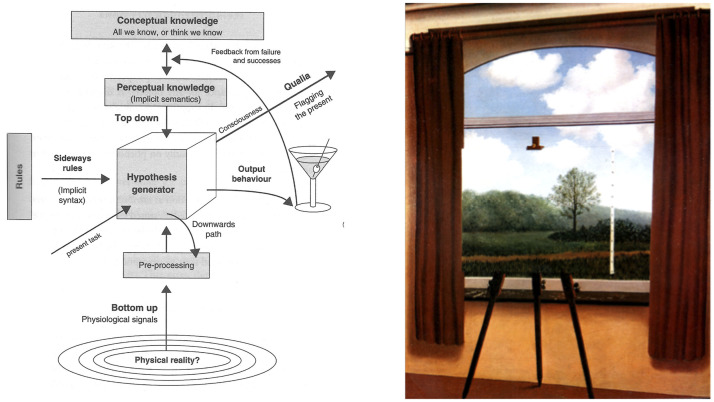
(a) *Rules* and *knowledge* in Gregory’s “Ins and Outs of Vision” (Gregory, 2009, Figure 1). (b) Magritte’s “The Human Condition” (La condition humaine).

As a consequence, it is assumed that there must be “higher-level” processes that involve inference, hypotheses, rule-following, and knowledge to account for the richness of our perceptions (Gregory, 1992, 2009; Rock, 1983; von Helmholtz, 1910/1962; see Figure 1a). A similar assumption of insufficiency can be seen today in the idea of “inverse optics.” Optics and geometry allow us to determine the characteristics of the projected image from the pattern of wavelengths reaching the eye, whereas there is considerably more uncertainty and ambiguity in determining the characteristics of the world that created a particular projected image. This ambiguity is most apparent in the field of 3-D vision. Rene Magritte’s famous painting “The Human Condition” (see Figure 1b) makes the point that a painting of a landscape on a flat canvas can create exactly the same retinal image as that produced by a real-world 3-D scene. Indeed, there is an infinite number of 3-D scenes—*equivalent configurations* (Ames, 1952, 1955; Ittelson & Kilpatrick, 1952; Runeson, 1988)—that could create the same retinal image.

## The History of Gibson’s Theoretical Position

### What Is Perception?

Traditionally, we have thought of perception as being about appearance—“*Why do things* ***look*** *as they do*” (my emphasis) as proposed by Koffka in his “Principles of Gestalt Psychology” (1935). Adherence to this definition is most clearly evident in the study of illusions—situations where appearance does not correspond to what is out there in the world (Gregory, 2009). Gibson, however, rejected this idea and asserted that “Perceiving is an achievement of the individual, not an appearance in the theatre of his consciousness. It is a keeping-in-touch with the world, an experiencing of things rather than a having of experiences” (Gibson, 1979 p. 239).

This idea makes sense if you consider the perceptual systems of other species. It seems very unlikely that flies or fish have *appearances* in the way that we do but they can certainly use information from their senses to guide their actions. And the same must be true for machine vision systems. This raises the (unanswerable) question of why we, as humans, have evolved to have conscious appearances and what their causal status is in controlling and guiding our actions. For Gibson, perceiving and acting are not separate processes but rather they are inseparable parts of what he referred to as a “perceptual system”—an idea that was the theme of his second book “The Senses Considered as Perceptual Systems” (1966).

In addition, Gibson’s idea of “keeping-in-touch” not only involves a completely integrated perceptual system but also the extraction of meaning—“*affordances*”—from sensory information. For Gibson, meaning is not something that is extracted or hypothesised at some “higher” stage of information processing, as is traditionally thought (e.g., Marr, 1982), but rather it is the essence of what it means to perceive. It seems unlikely that we would have evolved sensory and perceptual processes unless they provided us with information that allowed us to act appropriately, and this must hold for all species.

### Gradients and Optic Flow

In marked contrast to traditional theories of perception, Gibson argued that sensory information is rich and quite sufficient. In his first book “The Perception of the Visual World” (1950), Gibson sort to demonstrate this sufficiency through the related ideas of (a) gradients and (b) optic flow. These ideas were novel in 1950, but few people nowadays would disagree about their significance. Gibson regarded spatial gradients as important because the world we live in is made up of continuous surfaces rather than the isolated points and lines that were typical of previous research on perception. Surfaces create not only size and texture gradients but also disparity and motion gradients, which together provide us with rich information about the structure and layout of the world. It is important to remember that all these gradients are consequences of geometry and therefore quite independent of whether a visual system is able use this information. To a first approximation, objects that are twice as far away subtend angles that are half as large (Euclid’s law). As a consequence, the claim that gradients “provide information” is a statement about what a visual system *could use* rather than whether we, as human observers, are *able to use* that information; a point I shall return to later. But Gibson also wanted to challenge the assumption, which still dominates our thinking today, that perception is a hierarchical process starting with the extraction of simple “features” such as lines and edges and that these features are subsequently combined to generate object descriptions or representations, for example, Marr (1982). In this respect, Gibson’s thinking was clearly influenced by the ideas of Gestalt psychology that he learned from his mentor Kurt Koffka while at Smith College in the 1930s and 1940s.

The second important idea in Gibson’s “Perception of the Visual World” is that of optic flow—the patterns of motion in the optic array that are the result of observer or object movement. It should be remembered that prior to the 1950s, the overwhelming majority of perceptual experiments not only used simple line and dot patterns as stimuli but those stimuli were also presented for very brief intervals of time using tachistoscopes. Gibson’s insight was that we are constantly moving around in our environment and that our movements create lawful changes in the patterns of light reaching our eyes—optic flow.

**Figure 2.**
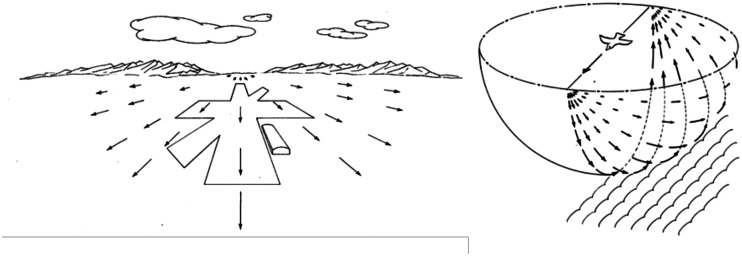
(a) Gibson’s depiction of the optic flow that would be produced as an aircraft approaches a landing strip. (b) Gibson’s depiction of the optic flow produced when a bird flies over a ploughed field.

The optic flow concept is important for several reasons. First, it provides a further example of the richness of the available sensory information for specifying the structure and layout of the surrounding world. Second, it has changed the way we study perception. I vividly remember Mike Braunstein’s demonstrations of structure from motion using 16-mm movie films in his presentations at the ARVO meetings in the early 1980s—a forerunner of present-day animations. Third, it provided the foundation for Gibson’s distinction between the optic array and the retinal image. In the “*Perception of the Visual World*” (1950), Gibson sketched the pattern of *outflow* that would be created as an aircraft approaches a landing strip (Figure 2a).

Note that Figure 2a and b represents two different ways of portraying optic flow. Figure 2a is an example of a projection of a 3-D scene onto a 2-D surface, such as a painting or a photographic image, whereas Figure 2b shows the projection of a similar scene onto a spherical surface. Because the distance from the centre of a sphere to all points on its surface is the same, the displacements on that surface are proportional to their angular size. This allows the magnitudes of angular size, direction, and motion speed to be visualised and directly compared in terms of their separations and displacements on that surface. There is no good reason for preferring one representation over the other, but there have been occasions when the characteristics of the two different representations have been confused (Frisby, 2009; Gillam, 2009; Rogers, 2009a).

### Optic Arrays and Retinal Images

There is, however, a very good conceptual reason for thinking about the sensory input in optic array terms. Traditionally, it has been assumed that the retinal image should be treated as the “starting point” for investigating perceptual processes. It is, after all, the location where the patterns of light from the world are transformed into neural signals. A good example of this assumption can be seen in the quotation from Gregory’s “Eye and Brain”: “We are given tiny distorted upside-down images in the eyes … ” (see earlier). In contrast, Gibson boldly asserted in his 1979 book that “The retinal image is not necessary for perception” (p. 61). What he meant by this provocative statement is not that we do not need eyes to see but rather that it is misleading to regard the features of the retinal image—its size, shape, pattern of wavelengths of light, and so on—as an appropriate description of the perceptual input. This idea was not new. One hundred and fifty years ago, von Helmholtz (1910/1962) wrote:

I am disposed to think that neither the size, form and position of the real retina nor the distortions of the image projected on it matter at all… .In the natural consciousness of the spectator the retina has no existence whatsoever. (pp. 166–167)

**Figure 3.**
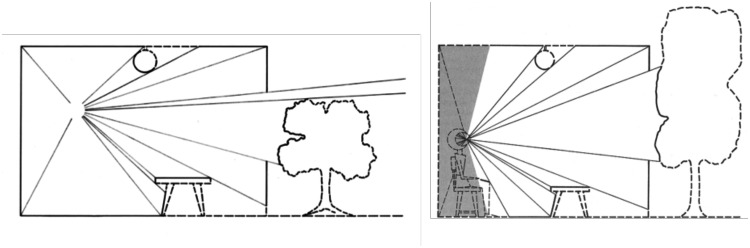
(a) Gibson’s drawing of the optic array at a particular vantage point. Note that the optic array extends over a full 360 deg of solid angle in contrast to the restricted *sample* of the optic array (b) that reaches the eye of a human observer (from Gibson, 1979).

So what is it about the optic array that makes it a better “starting point” (Rogers, 2007)? After all, the retinal image is merely the projection of the optic array onto the receptor mosaic. As mentioned earlier, the optic array is a description, in angular terms, of the patterns of light reflected off the multitude of the surrounding objects and surfaces from a particular viewing position or *vantage point*. Moreover, it is structured in the sense that smaller features are nested within larger structures such as the leaves on a tree (see Figure 3). But this is also true of the characteristics of the retinal image. What is different about an optic array description is that it is *independent* of any particular seeing machine—biological or man-made—that detects that pattern of light. As a consequence, identifying the characteristics of the optic array allows us to identify whether or not there is information available *in principle*.

The distinction between optic arrays and retinal images is best understood by taking a particular example—binocular stereopsis. It is often asserted that binocular stereopsis is based on the small differences or disparities between the images in the two eyes. But the information provided by binocular stereopsis is actually based on the differences between the optic arrays at two spatially separated vantage points. In other words, the disparity *information* exists independently of the observer and a particular visual system. As a consequence, the optic array description is also the appropriate starting point for creatures that do not have chambered eyes (where the lens projects an “image” onto an array of receptors). But more important, it allows us to separate out the question of whether or not there is available information (the computational theory) from the empirical question of whether and how we are able to use that information.

### The Transforming Optic Array

In “The Perception of the Visual World,” Gibson (1950) showed how the idea of an optic array description could be extended to include situations where the vantage point moves—the transforming optic array. In 1976, Jan Koenderink and Andrea van Doorn provided the mathematical proof that there is information in the transforming optic array to specify the 3-D shape of objects, which is quite separate from whether, and to what extent, human observers are able to use that information. In 1974, Nakayama and Loomis proposed a simple and physiologically plausible “convexity function” for extracting depth information from the velocity field (see also Frost & Nakayama, 1983). Their model was based on a concentric receptive field with antagonistic centre-surround organisation and directionally selective motion cells that are tuned to the *same* direction of motion. As a consequence, these hypothetical receptive fields are maximally sensitive to a *difference* between the amounts of motion stimulating the centre and surround—that is, directly analogous to the on-centre, off-surround simple cells identified by Hubel and Wiesel (1959) that respond best to a difference between the amount of light stimulating the centre and surround of a “simple cell.” Nakayama and Loomis point out that their “convexity detectors” would be “particularly sensitive to discontinuities of optic flow across the receptive field of the cell, independent of direction.” (p. 72)

**Figure 4.**
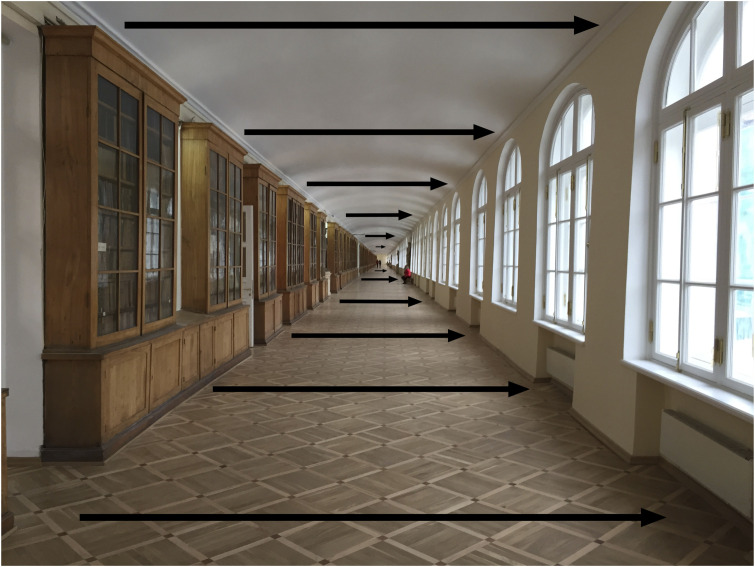
When the viewing position (or observer) moves from the right-hand side of the corridor to the left, optic flow is created. The amplitude of motion to the right is greatest for parts of the scene closest to the viewing position and least for parts of the scene farthest away.

There is a further conceptual point about Gibson’s spatial gradients and optic flow that is worth mentioning. Many perception textbooks classify the different sources of information about depth and distance into (a) primary and secondary (or “pictorial”) cues, (b) monocular and binocular cues, and (c) static and dynamic cues. What is often overlooked is that many of these different cues are based on the *same* underlying geometry. Take, for example, the photo of the 400-m long corridor in the Twelve Collegia University building in St Petersburg (see Figure 4). The arrows superimposed on the photo represent the patterns of motion created when an observer moves from the right-hand side of the corridor to the left-hand side. The motion amplitude is largest for parts of the floor (or ceiling) closest to the observer and least for parts of the floor farthest away, with a continuous gradient of motion in between.

But note that the gradient of *motion* exactly maps onto the gradient of the angular *size* from the parquet floor, which is not surprising because motion and size gradients are based on the same geometry. The size and motion gradients also map onto the *disparity* gradient between two images of the corridor taken from two spatially separated viewing positions (Rogers, 2017). And the same is true for any 3-D scene. I would argue that instead of treating these different sources of information as “independent cues” which need to be “combined” in the course of visual processing, it is better to see them as inextricably linked sources of information and reflected in the way our perceptual system operates.

### Assumptions

Gibson’s claim about the sufficiency of sensory information has been challenged by his critics on the grounds that we need to make *assumptions* about the world to extract perceptual information (e.g., Marr, 1982). For example, in both lightness and colour perception, it is argued that we need to make *assumptions* about the homogeneity (or at least the lack of discontinuities) of the illumination over the scene. Similarly, to be able to use the information provided by texture gradients, we need to *assume* the homogeneity of size of the texture elements. As humans, we are clearly able to make assumptions at a cognitive level, and hence the word itself suggests that some sort of “higher-level” process must be involved. But during the course of evolution, the illumination of scenes has been overwhelmingly homogeneous, and the sizes of the texture elements on surfaces have been overwhelmingly homogeneous, and therefore it should not surprise us that these characteristics of the world have been exploited during the evolution of our perceptual systems. Assumptions are not something that need to be *added*, in the way that they are in machine vision systems, but rather that they are an intrinsic part of an evolved perceptual system.

### Exterospecific and Propriospecific Information

**Figure 5.**
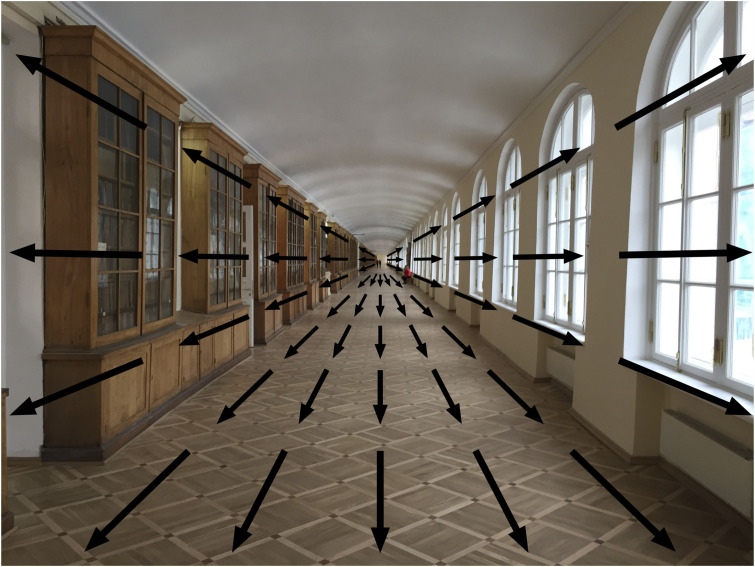
The arrows show the expansion pattern of motion that would be created if the viewing position (observer) moves towards the end of the corridor. This pattern of motion not only specifies that the viewing position has moved but also the direction and speed of that movement.

Traditionally, we think of the optic flow patterns that are created when we move around in the world as providing information about the structure and layout of objects in the 3-D world—*exterospecific* information. Gibson (1966), however, made the point that optic flow patterns also provide information about the movements of the observer—*propriospecific* information. Consider, for example, the pattern of optic flow created when we move through the 3-D world, such as walking down the St Petersburg corridor (see Figure 5). In optic array terms, there is a focus of expansion (a centre of outflow) in the direction of the observer’s movement towards the far end of the corridor, and the lengths of the superimposed arrows show the motion velocities of different parts of the corridor. This expansion pattern provides precise *propriospecific* information about the direction and speed of movement of the observer. Whether we, as human observers, are able to use this information is an empirical question that will be discussed later.

While it might seem sensible to make the distinction between exterospecific and propriospecific information, what is often important in our real-world activity is to obtain information about the relationship between our body, or parts of our body, with respect to the environment. Hence, Lee (1978) coined the rather clumsy term *expropriospecific* information “in an attempt to capture the relativistic nature of the information” (Lee, 1980: 170).

## The History and Legacy of Gibson’s Empirical Work

So far, I have discussed the history of Gibson’s conceptual ideas. In the following sections, I shall describe some of the empirical research that has been influenced by those ideas.

### Exterospecific Information: Information About the Layout and Structure of the World

von Helmholtz (1910/1962) famously described the retinal transformations produced by the movement of an observer in the following way:

In walking along, the objects that are at rest by the wayside … appear to glide past using our field view… .More distant objects do the same way, only more slowly… .Evidently, under these circumstances, the apparent angular velocities of objects in the field of view will be inversely proportional to their distances away; and, consequently, safe conclusions can be drawn as to the real distance of the body from its apparent angular velocity. (von Helmholtz,1910/1962, p. 295)

von Helmholtz’s description of the optic flow produced by observer movement is beautifully compelling, but it took more than a hundred years before Koenderink and van Doorn (1976), Ullman (1979), and Longuet-Higgins and Prazdny (1980) provided the mathematical proof that optic flow could, in principle, provide us with information about the structure and layout of the surrounding world. And longer still for there to be clear evidence that the human visual system is capable of using that information.

#### Motion Parallax

One of the first empirical studies of motion parallax was carried out by Bourdon (1902) who exposed two spots of light to a single eye in dark surroundings (see also Ono & Wade, 2005). The spots subtended the same visual angle but were at different distances. When the observer was stationary, the two spots appeared to lie at the same distance, but when the observer moved from side to side, the spots were seen at different distances. Similar experiments using a pair of spots were carried out by Eriksson (1973) and Degelman and Rosinski (1979). In 1984, Irvin Rock (1984) reported that observers were not able to make reliable judgements about the relative depth of nine individual discs of the same angular size located in three different depth planes. He concluded that “ … motion parallax does not by itself seem to be a cue to depth or distance” (p. 67).

**Figure 6.**
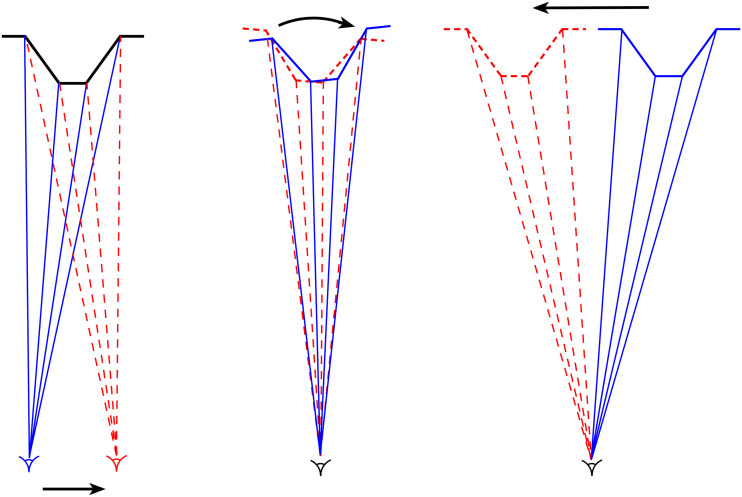
(a) When the eye of a monocular observer moves from left to right, the optic flow created by a pyramid-shaped surface is equivalent to that produced by a *clockwise* rotation of surface with respect to the observer’s line of sight (b). The same rotation of a surface with respect to the observer’s line of sight is created when the surface *translates* from right to left along a straight path in a frontal plane (c). Adapted from Rogers and Gyani, (2010).

In contrast to the early experiments using simple isolated stimuli, Gibson and his collaborators chose to investigate the motion parallax created by *surfaces*, which he referred to as *motion perspective* (Gibson et al., 1955, 1959). In their experiments, observers viewed a rear-projection screen on which there was a vertical velocity gradient of texture elements, created by a back-projected, 45 deg inclined surface that moved from side to side behind the screen. Most observers reported seeing a translating, *inclined* surface, although the opposite direction of inclination was seen by some observers. Note that unlike previous experiments, Gibson chose to investigate the motion perspective created by a surface that translated across the observer’s line of sight (object-produced parallax) rather than the motion parallax created by the side-to-side movements of the observer’s head (observer-produced parallax). In both cases, however, the parallax transformation is based on a small angle rotation of the surface with respect to the *line of sight* (see Figure 6; Braunstein, 1976; Rogers & Gyani, 2010).

**Figure 7.**
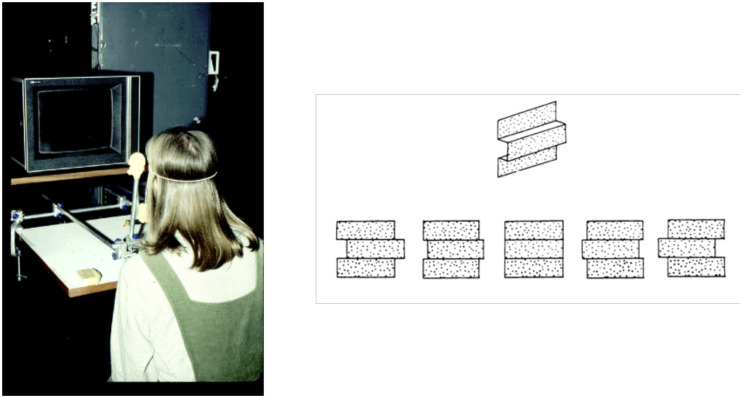
(a) A photo of our original motion parallax setup in which the monocular observer could make side-to-side head movements coupled to the transformation of the random dot pattern on the display screen. (b) The patterns of motion of the random dots as the observer moved from side to side while viewing a square wave corrugated surface.

And this is where my own experimental work on motion parallax is relevant. In 1977, an undergraduate student at St Andrews University—Maureen Graham—came to me to ask whether she could do her final year project on “optic flow.” At that time, I knew little about Gibson’s ideas and almost nothing about optic flow! I did, however, know something about binocular stereopsis and the work of Bela Julesz (1960, 1971) using random dot stereograms, and it occurred to me that we could design an analogous experiment to investigate motion parallax. The important feature of Julesz’s technique is that there is no information about the shape of the depicted 3-D structure in *either* of the binocular images alone—that information exists only after the binocular images^1^ have been compared.^2^ As a result, he showed that binocular disparities can be effective as an “independent cue for depth perception.”

In the motion parallax situation, we used a single random dot pattern on a display screen that appeared as a flat pattern of dots whenever the observer was *stationary* (see Figure 7). However, when the observer made small side-to-side head movements, that movement was used to transform the dot pattern on the display screen. The motion transformation mimicked the transformation that would have been produced by a real 3-D surface during a head movement (see Figure 5b). We reported that the observer saw a vivid impression of a 3-D surface, just like the impression seen when looking at a random dot stereogram (Rogers & Graham, 1979). To acknowledge Julesz’s influence on our thinking, we entitled the paper “Motion parallax as an independent cue to depth perception.”

In retrospect, I disagree with almost every word we used in that title (Rogers, 2009b). Our study was actually about motion *perspective* rather than motion parallax because what our observers reported was about the surface shape rather than simply depth. I also regard the word *cue* (with the connotation of uncertainly and ambiguity) as inappropriate. *Sources of information* is a clumsy but more appropriate label. Following Julesz (1960, 1971), we devised a technique that would show that observers could see the depth structure when motion parallax was the *only* source of 3-D information. This finding maybe important, but it ignores the fact that humans have evolved in a visual world where all the different sources of 3-D information have existed together. Isolated *cues* are the exception rather than the rule in the natural world, and therefore it seems to me that it is very unlikely that there are separate mechanisms whose outputs need to be *combined* at a subsequent stage of processing—as has been assumed in many cue combination models (e.g., Maloney & Landy, 1989).

One of the first researchers to exploit our observer-produced motion parallax technique was my good friend Hiroshi Ono and his colleagues at York University in Toronto (Ono et al., 1986, Ono & Ujike, 2005). They measured the extent of depth constancy as a function of viewing distance because the amount of parallax motion produced by a 3-D object is inversely proportional to the square of the viewing distance. They reported that parallax constancy was far from perfect and that at large distances the parallax-specified 3-D structure appeared to counter-rotate with the observer’s head movements (Ono et al., 1986). Several years later, Rivest et al. (1989) used both random dot patterns and familiar objects to examine the roles of familiar size, convergence, and apparent distance in the scaling process. They concluded that convergence information alone was insufficient to scale parallax motion, but it was effective when the convergence state affected the apparent distance to the surface.

In 1969, Gibson et al. highlighted the importance of the *dynamic* change of occlusion during head or object movement for specifying depth order. As an observer (i.e., the viewing position) moves from side to side, the texture of any more distant surface is progressively *concealed* (occluded) and *revealed*. Gibson himself not only argued for the importance of dynamic occlusion as a source of information about depth order (confirmed experimentally by the results of Kaplan, 1969), but he also claimed that dynamic occlusion provided evidence for the continuing *existence* of the occluded surface. He wrote:

The surface that was being covered was seen to persist after being concealed, and the surface that was being uncovered was seen to pre-exist before being revealed… .A sharp distinction was made between going out of sight and going out of existence. (Gibson, 1979, p. 190)

Ono et al. (1988) investigated the relative contributions of observer-produced dynamic occlusion and motion parallax in depth perception. In their experiment, a vertical bar covered with random dots was moved horizontally from side to side (linked to the observer’s head movement) and seen against a stationary background field of random dots. In the absence of head movement, there was no information to reveal the existence of the vertical bar to the monocular observer. However, when the observer moved from side to side, there was not only motion parallax to specify the depth order of the bar with respect to the background but also dynamic occlusion through the accretion and deletion of the background dot pattern. They discovered that the relative importance of two sources of information depended on the simulated depth separation of the bar and the background when the two sources of information were put in conflict. For small depth separations (< 25 arc min equivalent disparity), the perceived depth order was dominated by parallax information, whereas for large depth separations (> 25 arc min equivalent disparity), dynamic occlusion determined the depth order.

#### Classification of Optic Flow Transformations

**Figure 8.**
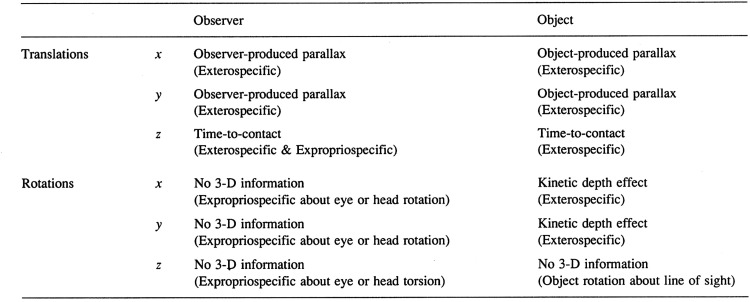
A modified version of Braunstein’s (1976) classification of the different optic flow transformations produced by either *translations* or *rotations* of either the *observer* or the *object* along or around the cardinal axes (from Rogers, 1993).

Motion parallax represents just one of the many different situations that create optic flow. In 1976, Mike Braunstein published an important monograph entitled “*Depth from Motion*” in which he not only described many of his own experiments on structure from motion but also presented a classification schema for the different optic flow scenarios (see Figure 8). Optic flow is produced when *either* the observer moves with respect to objects in the surrounding, stationary world *or* objects move with respect to the stationary observer. In addition, the observer’s eye or the object may either translate or rotate, and finally those translations or rotations may be around any one of the three cardinal axes: *x*, *y*, or *z*, where *z* is the depth axis. Translations of the observer along either the *x* (horizontal) or *y* (vertical) axis create *observer-produced parallax*, and translation of an object along either the *x* or *y* axis creates *object-produced parallax* (Braunstein, 1968; Braunstein & Tittle, 1988). All four of these situations provide exterospecific information about the 3-D structure of objects (see Figure 8). Note that the parallax motions are maximal for an observer moving in a direction *orthogonal* to the *z* axis (i.e., in the *x*-*y* plane), and there is an absence of parallax when the observer translates along the *z* axis. As a consequence, the region of zero parallax provides expropriospecific information about the direction of the observer’s heading (Cutting, 1986).

Rotations of an object around either the *x* (horizontal) or *y* (vertical) axis create the optic flow patterns that we refer to as the *kinetic depth effect* (KDE; Wallach & O’Connell, 1953). These rotations provide exterospecific information about the 3-D structure of the rotating object. Translations of either the observer or an object along the *z* (approach) axis provide exterospecific and expropriospecific information about the *time-to-contact* (TTC) of that object (Lee, 1974, 1980). The optic flow created by rotations of the observer’s eye or head around either the *x* or *y* axes provides propriospecific information about those eye and/or head rotations. Finally, the rotation of an object around the *z* (approach) axis provides no information about the 3-D structure of that object, but it does provide propriospecific information about the rotation of the observer’s eye or head around that axis.

#### Stereokinetic Effect

**Figure 9.**
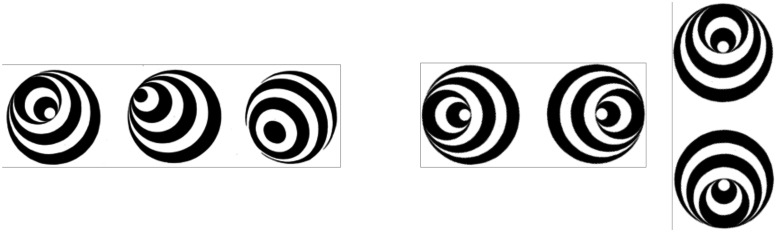
(a) Three examples of Stereokinetic Effect stimuli (from Wilson et al., 1983). The transformation of the pattern of eccentric circles is equivalent to that produced by a 3-D cone (a) that oscillates through a small angle around the *y* axis as in (b) and, with a 90 deg phase shift, around the (*x*) axis as in (c).

My assertion that the rotation of an object around the *z* (approach axis) provides no information about the 3-D structure of that object might seem to be incompatible with the results of experiments on the well-known *Stereokinetic Effect* (SKE) first reported by Musatti (1924). This effect can be seen when viewing a rotating flat disc with a pattern of eccentrically positioned circles or ellipses, such as those depicted in Figure 9a. Observers report seeing a cone- or ball-shaped 3-D form that “wobbles” around the *z* axis (between the observer and the disc; see Profitt et al., 1992; Wilson et al., 1983). The SKE works because the direction of motion of the black and white contours on the disc is ambiguous, like the contours of a rotating barber pole (the aperture problem). As a result, the eccentric contours appear to move inwards and outwards from the centre of the disc rather than to rotate with the disc. To a first approximation, the perceived inward and outward displacements of the contours of the rotating disc mimic the optic flow that would be generated by a 3-D object that oscillates to-and-fro through a small angle around both the *x* and *y* axes, but with the *x* and *y* axis oscillations 90 deg out of phase (see Figure 9b and c). As a consequence, the SKE should be considered as a variant of the KDE rather than as a separate perceptual phenomenon (see also Wallach et al., 1956). Evidence to support this interpretation comes from the observation that if random dots are added to the any of the patterns on the rotating discs in Figure 9a, the motion directions are no longer ambiguous. Instead, the cone- or ball-shaped 3-D form disappears, and the display is seen as a flat, rotating pattern.

#### Kinetic Depth Effect

Whereas motion parallax is the label given to the optic flow transformations created by *translations* of either the observer or 3-D objects along the *x* or *y* axes, the KDE is the label given to *rotations* of 3-D objects around the *x* or *y* axes (see Figure 8). Although there are many earlier reports of the perceptual effects created by rotating 3-D figures (e.g., Metzger, 1934; Miles, 1931), the first systematic study of these transformations was carried out by Hans Wallach and D. N. O’Connell in 1953. They used a shadow-casting technique in which the shadow of a 3-D wireframe or solid form was cast onto a rear-projection screen in front of the observer. They stressed that the wireframe or solid form should be placed as close as possible to the screen and that the distance between the light source and the object should be made large. In other words, the conditions represent an example of parallel (or orthographic) rather than polar projection. Consider an object rotating around a vertical (*y*) axis under parallel projection. Different parts of the rotating form oscillate back-and-forth *horizontally* in the projected image, but there are no changes in the *vertical* positions of the object’s features on the screen. Under these conditions, there is no information about the direction of rotation of object, and hence observers typically report that the direction of apparent rotation flips spontaneously. Under polar projection, which can be created in the shadow-casting situation by reducing the distance between the light source and the object, there are also changes in the vertical positions of features on the screen which Braunstein referred to as *vertical perspective* (Braunstein & Payne, 1968). Under polar projection conditions, observers typically report fewer or no flips in the direction of apparent rotation (Braunstein, 1962; Braunstein & Payne, 1968).

#### The Role of Vertical Perspective in Motion Parallax Transformations

The parallel-polar distinction is also relevant to the motion parallax situation described earlier. I argued previously that the optic flow transformation created by the translation of either the observer or the object in a frontal plane (i.e., motion parallax) is better thought of in terms of a small angle rotation of the object with respect to the observer’s line of sight (see Figure 6). If the angular size of the object is small (less than 5 deg), there will be little vertical perspective information to disambiguate the object’s direction of rotation, and hence observers should report depth alternations (flipping) as is typically seen in KDE demonstrations. However, Rogers and Graham (1979) reported no such ambiguity in their 20 deg × 20 deg displays. Why should this be? There are several possibilities. First, the vertical perspective changes^3^ in their 20 deg × 20 deg displays might have been sufficient to specify the direction of rotation of the object with respect to the observer’s line of sight (see Johansson & Borjesson, 1989). Second, given that their experiment was not carried out in complete darkness, there were visual cues from the ground plane and surroundings that could signal the observer’s location with respect to the surface and hence the direction of rotation. Third, there would have been proprioceptive and vestibular information about the position and movements of the observer’s head with respect to the object in the observer-produced parallax situation. Fourth, there could have been proprioceptive or outflow signals about the rotation of the observer’s eye during his or her side-to-side head movements that could signal the relative position of the observer’s head with respect to the object (Nawrot, 2003, Nawrot & Joyce, 2006; Nawrot & Stroyan, 2009).

In 1992, Rogers and Rogers attempted to dissociate the contributions of the first three of these possibilities. They found that all three sources of information—vertical perspective, visual cues from the ground plane, and proprioceptive/vestibular cues—all reduced the ambiguity of the perceived depth. In follow-up experiments, Rogers (2016a, 2016b) showed that the extraretinal information about eye position proposed by Nawrot plays little or no role in disambiguating the depth and direction of rotation of the 3-D surface. On the other hand, they showed that the vertical perspective information had a disambiguating effect even when the display subtended just 8.9 deg of visual angle.

### Expropriospecific Information

Expropriospecific information is information about the relationship between our body, or parts of our body, relative to the environment. Expansion patterns (see Figure 5) provide a good example of an optic flow transformation that could provide expropriospecific information about the relative movement between the observer and objects in the world. The use of expansion patterns as a source of expropriospecific information has been investigated in all four of the following situations (Lee, 1980): (a) the maintenance of balance, (b) perceived self-motion, (c) TTC, and (d) direction of heading.

#### Expansion Patterns and the Maintenance of Balance

**Figure 10.**
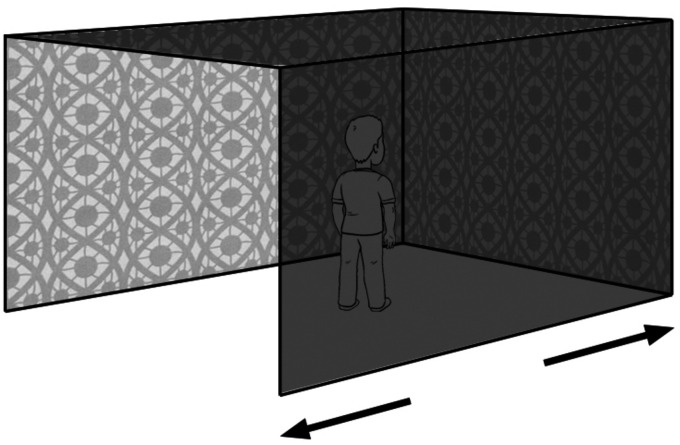
Lee’s observers stood in a suspended, swinging room which could move back-and-forth through a few centimetres.

Traditionally, it has been assumed that the way that we maintain our posture and balance is based on vestibular information from the otoliths and semicircle canals in the inner ear. This view was challenged by David Lee and his colleagues in the 1970s in their experiments using a “swinging room” (Lee & Aronson, 1974; Lee & Lishman, 1975). The room, which consisted of three walls and a ceiling suspended from above, could be made to swing back-and-forth through a few centimetres as shown in Figure 10. For the observer, who stood in the centre of the room, the swinging room created a pattern of optic flow that was very similar to that created by the observer’s own swaying movements. As the room swung towards the observer, the front and side walls create an expanding, outflow pattern of motion, and as the room swung away from the observer, the walls create a contracting pattern of motion. Lee and colleagues reported that their observers swayed back-and-forth in phase with the room’s movements—according to Lee’s words, they were “*hooked like puppets.*”

Importantly, the observers never realised that they were swaying, thereby demonstrating how optic flow can influence behaviour without the observer’s awareness. However, Lee’s ingenious experiment is not without its problems. The optic flow produced by an observer’s movements is dependent on the distance of surfaces from the observer. To appreciate this, think about the transforming optic array in terms of an array of strings connecting the surrounding surfaces to the observer’s eye. Close surfaces create the largest angular changes in the strings at the eye (and hence the optic flow) when the observer moves, whereas distant surfaces create the smallest angular changes. In Lee’s swinging room, the front wall was only 30 cm away from the observer and side walls just 80 cm on either side, thereby maximising the chances of optic flow affecting balance. We also need to know whether the optic flow created by observer sway in a normal-sized room helps us to maintain balance. In addition, the swinging room had no floor and because humans spend most of their lives standing on a ground plane surface, it is also important to determine whether the optic flow created by the ground plane surface plays a role in the maintenance of balance (see Rogers et al., 2007).

#### Expansion Patterns and Perceived Self-Motion

One important feature of Lee’s experiments on balance is that observers were quite unaware that they were swaying in synchrony with the movements of the swinging room. But what happens when the amplitude or velocity of the back-and-forth movements of the room is increased? The answer is that the observers feel that they are moving—self-motion or vection. In a clever experiment, Lishman and Lee (1973) investigated perceived self-motion in their swinging room to discover the relative importance of visual and non-visual information. To do this, they asked observers to take a couple of steps forward in the swinging room. In this case, both visual and non-visual information signal the observer’s forward movement. But Lishman and Lee linked the observer’s forward steps to the movements of the room in such a way that the room moved through *twice the distance* in the direction of the observer’s steps. This had the effect of creating a *contracting* pattern of optic flow at the observer’s eye—signalling that the observer was moving *backwards*. In this condition, observers reported that they felt they were moving backwards. In other words, visual information from optic flow overrides the non-visual, vestibular, and proprioceptive information.

**Figure 11.**
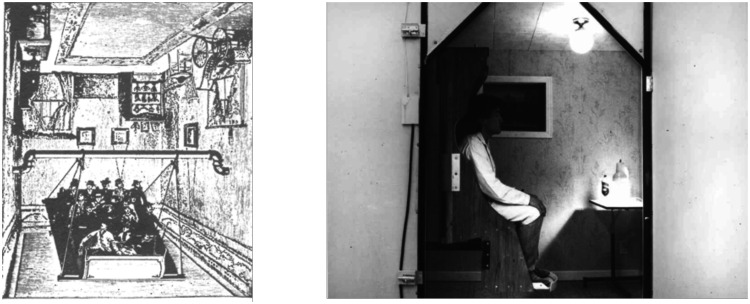
(a) A picture of a 19th century “haunted swing” fairground ride. (b) Ian Howard’s “Tumbling Room.”

The study of vection predates Lee’s swinging room experiments, from the “haunted swing” fairground rides in the 19th century (Figure 11a) to Gunnar Johansson’s “Elevator illusion” (Johansson, 1977) and Ian Howard’s “Tumbling Room” (Allison et al., 1999; Howard & Childerson, 1994; Figure 11b). The results of all these studies show both the importance and dominance of optic flow in our perception of self-motion. However, the situation is not quite as straightforward as it may seem. Allison et al. (1999) showed that *more* observers experienced the feeling of tumbling (head over heels) in their furnished room (Figure 11b) than in a large sphere covered with random dots that rotated around the observer. They argued that the room provided additional “frame” and “up-down” information, and this affected the observers’ perception of self-motion, in addition to the optic flow.

#### Expansion Patterns and TTC

Consider the approach of an object towards us, such as a tennis or cricket ball. As the ball approaches, its angular size in the optic array—its *rate of dilation*—increases (Lee, 1980). If the object’s approach is at constant velocity, there is a simple inverse mathematical relationship between the rate of dilation of the image and the time before the object reaches the observer—referred to as the *TTC*. Lee proposed that the inverse of the rate of dilation—(τ)—could provide observers with an estimate of the actual TTC. Hence, observers could potentially use the instantaneous value of tau of the approaching object to estimate of how soon that object will reach them. This is important in many sports where players need to be able to start closing their hands or moving their racket or bat before the ball actually reaches them. The feature that makes this computational theory attractive is that the rate of dilation is independent of both the object’s distance from the observer and its approach velocity. Moreover, the rate of dilation of a particular image feature would be relatively easy to recover in terms of a possible neurophysiological mechanism (Koenderink, 1986). There is also an important conceptual point. Lee (1980) argued that while we typically think about visually guided behaviour in terms of estimating depth and distance in a 3-D world, there are many situations in which it is *timing* rather than distance that provides the relevant control parameter (Lee & Reddish, 1981).

**Figure 12.**
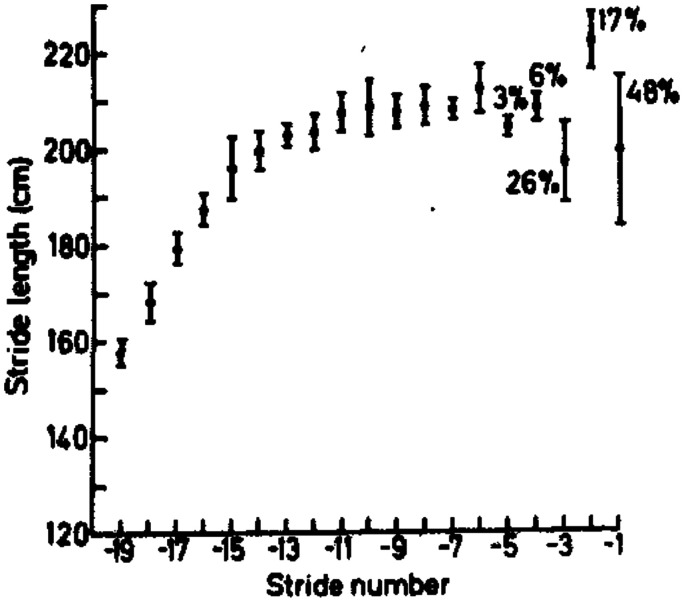
One athlete’s results in Lee et al.’s (1977) study. They show how stride length increases as the athlete approaches the takeoff board in a consistent, stereotyped way (over many trials). However, in the final few strides prior to takeoff, the athlete’s stride length varies considerably, indicating that the athlete was making changes in her stride pattern.

Estimation of TTC is not just important for judging how soon a moving object will reach the observer—it is also important for judging how soon the observer will reach a location in the surrounding visual world. Walking, running, and driving towards a target or destination are obvious examples (e.g., McLeod & Ross, 1983). In an important early paper, Lee et al. (1977) investigated how professional long jumpers controlled their stride patterns in their run up towards the takeoff board. The long jumpers themselves thought that their stride pattern was predetermined, as a result of extensive practice, but Lee et al. showed that the long jumpers were actually adjusting their strides in the final few steps before takeoff (see Figure 12). Moreover, they argued that the important feature that the athletes could control was the upward force they exerted on each stride because that would affect the *duration* of their strides, independently of how fast they were running. Hence, instead of adjusting the *length* of their strides to fit in with the remaining *distance* to the takeoff board, Lee et al. argued that the long jumpers were adjusting their upward force (and thus the *time* for each stride) to fit in with the remaining *time* until they reached the board.

Lee et al.’s results and analysis sounds very convincing, but because the study was purely observational, it is impossible to rule out the possibility that the athletes were adjusting their strides using distance rather than timing information. However, in many subsequent experiments, Lee and his colleagues have provided convincing evidence that many different species, including gannets and humming birds, use the rate of dilation to control their behaviour (see also Warren, 2021). In addition, Wang and Frost (1992) showed that neurons in the pigeon nucleus rotundas were sensitive to the rate of dilation of the image of an approaching ball, independent of the ball’s size and speed.

#### Expansion Patterns and Direction of Heading

The expanding optic flow pattern created when an observer moves provides a fourth example of potential exterospecific information. According to Gibson, the visual surroundings produce an outflow pattern of motion that radiates from a “focus of expansion” (see Figure 5). In other words, there is a singularity in the flow field that lies in the direction of motion of the vantage point. Gibson depicted this in his 1950 book using the outflow pattern that would be produced when a plane is heading towards a landing strip (see Figure 2a).^1^ One significant advantage of using optic flow to guide behaviour for any species is that the information is reliable and unaffected by side winds or water currents. This means that even though you might head (i.e., orient your body) towards a particular target when swimming across a flowing river, the focus of expansion will tell you the actual point to which you are heading. This will also be the case when attempting to land a plane on a runway when there is a cross wind.

There are, however, two significant problems with the use of optic flow. First, there is the practical problem of how the locus of that focus could be extracted from the optic flow field and second, the conceptual problem that we have access to only the retinal image, not the optic array. In particular, eye movements alter where the image of the surrounding world projects to the retinal mosaic. To get around this problem, Royden et al. (1992) proposed that if we had access to eye position information, we could, in principle, use that information to recover the focus of expansion in the optic flow field and hence determine the heading direction. Their experimental results suggest that this might be possible.

Koenderink and van Doorn (1981) proposed an alternative strategy to deal with the same problem. They showed that the optic flow field could be decomposed into two components: (a) the overall translations of the flow field on the retina (caused by both eye and head movements), which they referred to as the *solenoidal flow* and (b) the differential characteristics of the flow field which they referred to as the *lamellar flow* (see also Longuet-Higgins & Prazdny, 1980). Cutting (1986), however, has suggested that such a strategy might not work if the observer is moving along a curved path (see Land & Tatler, 2009 for a more detailed discussion of this issue). In 1982, Regan and Beverley argued that because the focus of expansion in the retinal image was in the direction of gaze rather than in the direction of heading, observers might use the *point of maximum rate of magnification*. However, as Cutting (1986) pointed out, there are many problems with this proposal both terminological—magnification refers to the equal enlargement of the image—and practical in terms of the experimental evidence that Regan and Beverley presented to support their idea.

Much more promising is the idea of detecting the region of zero parallax (Cutting, 1986). As mentioned previously, motion parallax is *maximal* in directions orthogonally to the direction of heading and *zero* in the direction of heading, and, importantly, this is true whether the observer is looking in the direction of heading or looking in some other direction. In the limit, detecting the zero parallax in the direction of heading corresponds to the task of detecting a change in the vernier alignment of objects close to the direction of heading (Telford & Howard, 1996).

The idea of using optic flow to guide heading behaviour is very appealing, but it overlooks the fact that there is also static (non-flow) information that might also be used. To walk, run, or swim towards a given target, humans are capable of aligning their bodies in the direction of that target before they start moving. Not only are we able we rotate our heads to *face* a target (i.e., head-centric localisation), but we can also rotate to align our bodies with a target (ego-centric localisation; Howard & Templeton, 1966). And this could also be done continually *while* we are walking, running, or swimming. In other words, ego-centric *visual direction* provides an alternative source of heading information to optic flow. In the case of driving a car, it is also easy to determine the direction in which the car is heading because this is given by a particular location on the windscreen, assuming the car is not drifting or spinning.^4^

How can we decide whether we use (dynamic) optic flow or (static) visual direction information for any particular task and circumstances? A simple displacing prism in front of the eye provides a possible answer. Displacing prisms change the apparent position of the target on the retina—strictly speaking they create a *change of eye position* to keep the image on the fovea—but they do not change the properties of the differential flow field. When an observer wearing displacing prisms is asked to walk towards a visual target, it has to be true that they start off walking in the visual direction of the target—that is, heading to one side of the target and at an angle that corresponds to the displacement angle of the prism. If the observer continues to be guided by visual direction information, it is easy to show that they should approach the target along a *curved* path that ultimately leads them to the target.

Several studies have used this paradigm, but the results are contradictory. The experimental data from Rushton et al. (1998), Rogers and Dalton (1999), Harris and Carre (2001), and Harris and Bonas (2002) all show that locomotion is primarily guided by the visual direction of the target rather than by information in the optic flow field. In contrast, the results and conclusions from the Warren et al. (2001) study are somewhat different, as were the methods used. Using a virtual reality headset rather than displacing prisms, they showed that the characteristics of the surrounding visual field play an important role. When an observer was asked to approach a single *target line* in an otherwise dark environment, the observer walked along a curved path that was close to that predicted by the visual direction hypothesis. On the other hand, when the texture of the ground plane was visible, observers walked along curved paths that were substantially less curved than predicted by the visual direction hypothesis. These and subsequent results by Warren and his colleagues suggest that the locomotor control used for tasks such as walking or running is a product of both optic flow and visual direction information, with the balance between the two sources of information dependent on the salience of the ground plane texture and the parallax generated by objects standing on the ground plane surface. A more extensive discussion of this issue is given in Warren (2021). This conclusion should not surprise us. It seems more than likely that our perceptual systems have evolved to use whatever information is available in a particular situation. As Cutting concluded in 1986:

An observer moving through an environment has a choice of invariants to use to guide his or her way… . It seems probable, in fact almost certain, that this may be the general perceptual situation, given a rich environment or even a rich experimental setup. (Cutting, 1986)

## Summary and Conclusions

### Conceptual Legacy

There seems to be little doubt that Gibson’s ideas have influenced the thinking of a many researchers in the field. The idea that the primary function of perception is to allow us to act (in the most general sense) is widely accepted. Humans, and other species, would never have evolved sensory mechanisms if those mechanisms didn’t allow a species to act appropriately. Moreover, it seems likely that perception and action constitute an integrated perceptual system rather than two separate and relatively independent modules. In my view, the identification of two separate neural pathways in the brain—the ventral and dorsal streams—has been somewhat misleading in this respect. That is not to say that those pathways don’t exist or that different parts of the brain might not be more or less involved into different aspects of functioning of the perceptual system but rather that it is premature to divide up “perceptual processing” into different modules and attribute different functions to those modules until we have a better understanding of what the brain is trying to do. Richard Gregory (1961) made this point many years ago in his paper “The Brain as an Engineering Problem.”

Gibson also argued strongly for the idea that perception is about the relationship—the *synergy* or *mutuality*—between the perceiver and the environment. He pointed out that when we describe the characteristics of the perceiver’s perceptual system, we are effectively describing the characteristics of the animal’s particular environment and likewise when we describe the characteristics of the particular environment, we are effectively describing the characteristics of the perceiver’s perceptual system. In other words, we can only describe the features of the world that we perceive—its colours, textures, and motions—through the constraints and limitations of our particular perceptual systems. As a consequence, he argued that perception isn’t solely about the perceiver; rather, it is about the *relationship* between the perceiver and the environment.

In 1979, Gibson had little or nothing to say about the physiological basis of the perceptual system, and hence it is interesting to speculate on what he would say today in the light of the enormous advances that have been made in this field. Certainly, he regarded the psychological and the physiological as different levels of explanation—each valuable in its own right—but at the same time, he doubted whether results in either domain could constrain ideas in the other. A similar reservation was expressed by David Marr in 1980 when he wrote: “Modern neurophysiology has learned much about the operation of the single nerve cell but disconcertingly little about the **meaning** of the circuits that they compose in the brain” (p. 199, my emphasis).

One of the fundamental features of Gibson’s position was his claim about the richness of sensory information. Traditionally, we have thought of memory and cognition as *supplementing* the paucity of sensory information. But it seems to me that either there is something in the sensory information that allows us, for example, to recognise or distinguish between faces or there isn’t—in which case memories or cognition can’t help (see Michaels & Carello, 1981). We don’t imagine that to distinguish between colours we need to match the visual input to some stored memory of that colour: The output of our trichromatic system *is* the colour. Gibson would have argued that to distinguish faces we need to have an “attuned” perceptual system resulting from evolution and a lifetime of perceptual experience. We don’t have to *remember* how to ride a bicycle, but we do need to have an attuned perceptual-motor system. Gibson’s (1979) metaphor of an attuned system was a radio receiver that would “resonate” to different radio frequencies. If he were alive today, I’m sure he would have preferred a deep learning network as a more appropriate model in which the network weights are modified as a result of experience.

However, the ability to distinguish faces or colours is not what Gibson regarded as the reason why we have a perceptual system: They merely contribute to what he argued was the essential function of perception—the extraction of meaning (affordances). Meaning, in the Gibson’s affordance sense, is not something that is added on afterwards as it is in Marr’s (1982) model of perception. Rather, it is the essence of perception because meaning is what allows us to function appropriately in the world. This is more convincing if you consider the perceptual systems of other species: A bird needs to choose where it should nest, what is the best food to eat, and who is the best individual to mate with. To accomplish these tasks, there must be sufficient information in the patterns of light reaching the bird’s perceptual system. There is a paradox here. We are content to accept that there is information from texture gradients, disparity, and parallax to specify the physical characteristics of the ground plane surface—its flatness, texture, and roughness—but we are reluctant to accept that there could be information to indicate whether that surface can be walked on. But what *can* tell us that a particular ground plane surface can be walked on? Our memories, our knowledge? If the sensory information wasn’t there in the first place, how could it be stored in memory or added to our knowledge? Does a herring gull chick need to have a memory or knowledge that a red spot is the place to peck to obtain food? (Tinbergen, 1953).

However, despite the intuitive appeal of the affordance concept, it has proved very difficult to test the concept experimentally (but see, e.g., Warren, 1984). In my view, the most important legacy of Gibson’s (1979) book is that it has prompted us to question and challenge our underlying assumptions about the nature of perception and the functions of the perceptual system, whether or not we agree with what he said.

### The Empirical Legacy of Gibson’s Ideas of Optic Flow

Empirical studies of the patterns of motion created by our own movements and the movement of objects in the world have a long history—von Helmholtz’s (1910/1962) astute observations about the power of motion parallax and Wallach and O’Connell’s (1953) studies of the KDE provide just two examples. Since the publication of “The Ecological Approach to Visual Perception” in 1979, there has been a dramatic increase in the number of researchers and the number of research papers on topics related to the idea of optic flow. These include the topics discussed previously such as structure from motion; motion parallax; maintenance of balance; the perception of self-motion; TTC and direction of heading; and many others. The influence of his ideas is also very evident in the content of presentations in the major vision conferences. At the first ARVO meeting I attended in 1980, there was only one paper session labelled “Motion and Depth” and only one presentation (ours) that could be labelled as “optic flow.” In contrast, the use of optic flow for guiding and controlling a whole range of behaviours in humans and other species has featured prominently in both VSS and ECVP meetings over the past 30 years.

It is difficult to assess what proportion of the authors of these papers and presentations were directly influenced by Gibson’s conceptual framework, and possibly it does not matter. The increase is partly due to improvements in technology—vastly more powerful computers, improved displays including head-mounted systems, and other virtual reality techniques. These improvements have played an important role in allowing researchers to investigate aspects of perception and perceptual-motor behaviour that would have been impossible 40 years ago. At the same time, the importance of dynamic change in the perceptual input, and especially of optic flow, is now accepted and appreciated over a much wider field of perception. For example, after many decades of research on face recognition and the perception of emotion using single static images (often presented tachistoscopically), there is now good evidence that the dynamic changes of face features play an important role in face perception (Jack, 2019).

There is, however, a danger of accepting some of Gibson’s ideas uncritically. It is quite appropriate to explore the potential richness of the sensory information and to attempt to discover which aspect of that information might underlie the control of a particular behaviour, for example, Lee’s optic parameter “τ” as a basis for estimating TTC. However, it is easy to fall into the trap of designing an experiment to test this possibility without testing other alternatives. As Donald Broadbent pointed out in his 1973 book “In Defence of Empirical Psychology,” a good experiment is one that not only provides results that are compatible with the experimenter’s own hypothesis but also provides results that are incompatible with alternative hypotheses. There is the further danger of assuming that there is just one source of information to guide a particular action. As a result of evolution, it seems more than likely that all animals have been able to exploit whichever source of information, or combination of sources, is appropriate for guiding a particular activity in a particular situation. It seems very unlikely that there is going to be a single correct solution that applies in all cases and under all circumstances.

In conclusion, it seems to me that Edward Reed’s (1998) assessment of Gibson’s legacy is still valid today: “whatever the final status of Gibson’s theories, his work has significantly changed the scientific study of human awareness and has deep implications for anyone interested in human behaviour and knowledge.” (p. 2)

## Notes

1 John Mollon (1997) has provided evidence that Gibson was aware of the Cambridge psychologist, G. C. Grindley’s, earlier (secret) report entitled “Notes on the perception of movement in relation to the problem of landing an aeroplane.” In that report, Grindley sketched the pattern of motion vectors that would be produced when a pilot was gliding towards a landing strip. The sketch bears a strong resemblance to Gibson’s own diagram that was first published in a mimeographed report of 1947 (see Figure 2a).

2 Note that to extract disparities, features of the two images need to be *compared*. Nothing needs to be “combined” or “fused.”

3 The motion perspective changes in Rogers and Graham’s motion parallax experiment are created because of large size of their display (20 × 20 deg). As the observer moves from side to side, the *obliqueness* of depicted surface (with respect to the observer) changes over time, whether the surface is flat or corrugated. It is the changing trapezoidal shape of the overall pattern of random dots that provides the vertical perspective information in their experiment. This dynamic change in the parallax situation is directly analogous to the static difference between the two binocular views of a similar surface that Rogers and Bradshaw (1993) called differential perspective.

4 Formula 1 cars are now fitted with a “halo” safety structure that provides an excellent reference point for judging where the car is heading.

5 This article is based on a presentation given at the 2019 ECVP symposium entitled “The Ecological Approach of James J. Gibson: 40 years later” (Rogers, 2019).
